# Multimodal cantilevers with novel piezoelectric layer topology for sensitivity enhancement

**DOI:** 10.3762/bjnano.8.38

**Published:** 2017-02-06

**Authors:** Steven Ian Moore, Michael G Ruppert, Yuen Kuan Yong

**Affiliations:** 1 The School of Electrical Engineering and Computer Science, The University of Newcastle, Callaghan NSW 2308, Australia

**Keywords:** atomic force microscopy, multifrequency AFM, multimodal AFM, piezoelectric cantilever, self-sensing

## Abstract

Self-sensing techniques for atomic force microscope (AFM) cantilevers have several advantageous characteristics compared to the optical beam deflection method. The possibility of down scaling, parallelization of cantilever arrays and the absence of optical interference associated imaging artifacts have led to an increased research interest in these methods. However, for multifrequency AFM, the optimization of the transducer layout on the cantilever for higher order modes has not been addressed. To fully utilize an integrated piezoelectric transducer, this work alters the layout of the piezoelectric layer to maximize both the deflection of the cantilever and measured piezoelectric charge response for a given mode with respect to the spatial distribution of the strain. On a prototype cantilever design, significant increases in actuator and sensor sensitivities were achieved for the first four modes without any substantial increase in sensor noise. The transduction mechanism is specifically targeted at multifrequency AFM and has the potential to provide higher resolution imaging on higher order modes.

## Introduction

The invention of the atomic force microscope (AFM) [1] provided for the observation of the nanoscale like no other tool before it [2]. The technologies derived from research into the AFM have led to developments in nanomachining [3], nanometrology [4], material science [5], semiconductor manufacturing [6–7] and high-density data storage systems [8–10].

The AFM uses a sharp probe tip at the free end of a cantilever to interrogate and image the surface of a sample [11–13]. When using the AFM in dynamic mode [14], the cantilever is excited at its fundamental modal frequency and the probe lightly taps the surface of the sample. Observed changes in the amplitude, phase or frequency shift of the cantilever’s motion correlate to properties of the sample [15]. When closing a feedback loop around these observables with the *z*-axis nanopositioner, the controller output is routinely used to map the surface topography of the sample. Recently, the additional excitation and detection with multiple frequencies has led to vast improvements in the nanomechanical characterization of the sample beyond it’s topography [16]. For these multifrequency AFM (MF-AFM) methods, higher order modes provide enhanced imaging properties such as higher modal stiffnesses and faster response times. It was shown that these higher modes can be more sensitive to material properties such as elastic modulus and damping coefficients [17–19]. Additionally, stiff cantilevers have proven to provide high resolution imaging in ambient and liquid environments using quartz resonators [20–21].

Traditional AFM cantilever instrumentation requires a piezoelectric stack actuator at the base of the cantilever for excitation [3] inevitably adding additional resonances as is visible from the so called forest of peaks [22]. These additional frequency components make cantilever resonance tuning almost impossible in liquids [23–24] and can alter the cantilever response rendering the identification and subsequent analysis of higher modes exceedingly difficult. For this reason, numerous integrated actuation methods such as magnetic [25], photothermal [26], resistive thermal [27], ultrasonic [28] or via a piezoelectric layer [29] have been devised.

In order to measure the cantilever deflection, the optical beam deflection (OBD) method [30] is still the widely used standard. However, the measurement setup for the OBD method has limitations, such that it requires frequent laser alignment, a cantilever with a reflective surface and a certain minimum dimension as dictated by the laser spot size. Further, the method suffers from imaging artifacts due to optical interferences originating from stray light reflected by the sample surface [31–32] and bandwidth limitation of the readout circuit [33]. In contrast, a strain-based deflection measurement offers several advantages including a much more compact measurement setup, potential for scalability to cantilever arrays as well as increased sensitivity for smaller cantilever dimensions [34–38].

Among the existing integrated actuation and sensing methods, piezoelectric transduction seems to be the only one capable of simultaneously serving as an actuator and a sensor even with a single active layer [39–40]. A set of cantilever designs exist which integrate a piezoelectric transducer onto the cantilever as part of the microfabrication process [41]. While good imaging performance is achieved when operating these cantilevers at higher order modes, the topology of the piezoelectric layer is designed with no consideration of the modal response of the cantilever.

A number of researchers have investigated shaping the piezoelectric layer to actuate or sense a single mode of beam and plate structures whilst filtering the responses of the other modes [42–46]. The design of these transducers, denoted modal sensors/actuators, encompasses the modeling and modal analysis of the structure in order to determine the modal frequencies and deflection mode shapes. Here, the overall deflection is the linear combination of mode shapes which have a fixed spatial distribution. Moreover, there exists a linear mapping from the deflection of the structure to the charge developed on the piezoelectric layer and hence the charge response is related to the spatial distribution of each mode shape. If the mode shapes are orthogonal, an analytical approach can be used to shape the piezoelectric layer. Otherwise optimization is used to shape the piezoelectric layer to minimize response to the undesired modes.

This work formulates the design of the topology of the piezoelectric layer on an AFM cantilever to maximize the actuator gain and sensor sensitivity with respect to the cantilever’s higher order modes. Compared to previous work on modal sensor/actuators [42–46], the design specification of the presented work is to enhance the desired modes rather than suppress the undesirable modes. This difference leads to fundamental changes in the design strategy, resulting piezoelectric layer topology, instrumentation, actuator characteristics and sensor characteristics. Indeed, the justification for this altered approach comes from the fundamental reasons for multifrequency AFM which is based on the assumption that additional information is encoded in these higher modes. To enable the optimization of the piezoelectric response to higher order modes, plate theory with finite element analysis is used to determine the spatial distribution and polarity of the transducers response for a given mode shape. Using this result, the piezoelectric layer is split into isolated regions whose individual responses constructively combine in order to maximize the actuator gain/sensor output.

The remainder of the paper is organized as follows. Section ’Modal analysis of the piezoelectric cantilever’ outlines the modeling approach to determine the spatial charge distribution of the piezoelectric transducer as a function of its modal response. In section ’Proposed piezoelectric cantilever designs’, results of this analysis are used to determine the design of the piezoelectric actuator arrangements to maximize the transducer response for the first four modes of a cantilever with a stepped geometry. In section ’Instrumentation of the cantilever’ the working principle and modeling of the instrumentation is presented. The experimentally determined actuator and sensor transfer functions are presented in section ’Experimental Results’, which highlight the actuator gain and sensor sensitivity improvements of the proposed designs. In addition, this section presents and discusses the noise characterization of the sensor. The cantilever designs presented in this work target a single mode each. Section ’Instrumentation for multifrequency AFM’ outlines a method to design and instrument the cantilevers to target multiple modes.

## Results and Discussion

### Modal analysis of the piezoelectric cantilever

**Figure 1:**
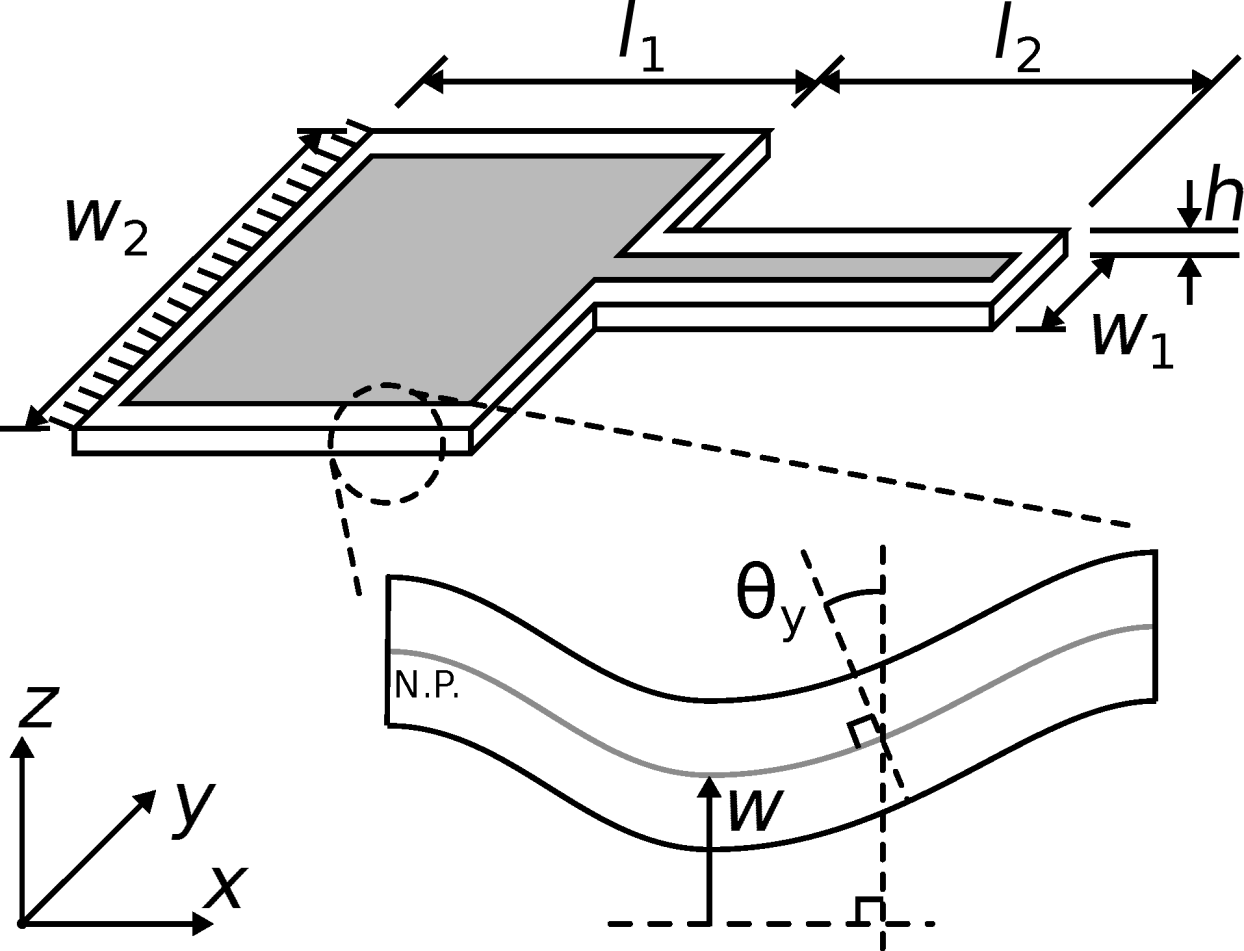
The dimensions of the basic cantilever design. The gray zone is the piezoelectric layer. The zoomed in section of a plate shows the out of plane deflection *w* and the rotation of the normal of the cantilever’s neutral plane (N.P.) about the *y*-axis θ*_y_*. The rotation of the normal of the cantilever’s neutral plane about the *x*-axis θ*_x_* is equivalent to θ*_y_* for a section in the *yz*-plane. The fixed boundary of the cantilever is shown to the left of the image.

Figure 1 shows the silicon cantilever analyzed in this work. The dimensions in the diagram are stated in Table 1. The benefit of the stepped geometry of the cantilever is that higher modes are more closely spaced compared to rectangular cantilevers [47–48] and higher mode deflections are amplified [49] which benefits higher harmonic/higher mode applications [41].

**Table 1:** Cantilever dimensions shown in Figure 1.

Parameter	Value

*l*_1_	400 μm
*l*_2_	400 μm
*w*_1_	100 μm
*w*_2_	500 μm
*h*	10 μm

The cantilever is modeled using Mindlin plate theory and a finite element (FE) model is developed to perform modal analysis [50–51]. Modal analysis using the FE model provides a solution to the out-of-plane deflection *w*(*x*,*y*,*t*) and the rotations of the normal of the cantilever’s neutral plane around the *x*-axis and *y*-axis, θ*_x_*(*x*,*y*,*t*) and θ*_y_*(*x*,*y*,*t*) respectively. These quantities are shown in Figure 1. Assuming a thin piezoelectric layer, the response of the piezoelectric transducer is proportional to the strain at the surface of the cantilever. The in-plane strains at the surface of the cantilever are [51]

[1]
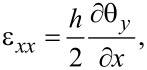


[2]
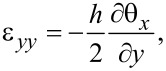


[3]
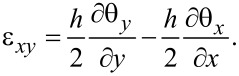


The electrodes are uniformly distributed on both sides of the piezoelectric layer to generate electric fields only in the *z*-axis. Therefore, the electric displacement in the piezoelectric material is [52]

[4]
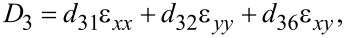


where *d*_31_, *d*_32_ and *d*_36_ are the piezoelectric coefficients. Assuming the piezoelectric material is poled along the *z*-axis and is homogeneous, the coefficients *d*_31_ = *d*_32_ = *d* and *d*_36_ = 0 [52–53]. The charge produced is the integral of the electric displacement, that is

[5]
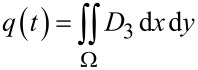


[6]
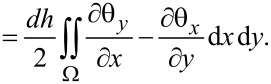


The domain Ω is the area of the piezoelectric layer.

Modal analysis with the FE model evaluates harmonic solutions for θ*_x_* and θ*_y_* of the form

[7]
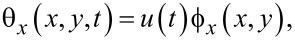


[8]
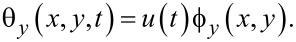


The spatial functions 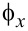
(*x*,*y*) and 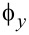
(*x*,*y*) are the mode shapes of cantilever.

In the following analysis, the domain Ω from Equation 5 is restricted to the domain *A_e_* of a single rectangular element from the mesh used in the FE model. The modal analysis calculates the rotations 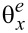 and 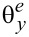 at the four nodes of the element. The mode shapes over an element are

[9]
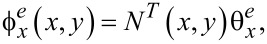


[10]
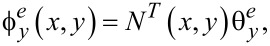


where *N*(*x*,*y*) are the shape functions [50–51]

[11]
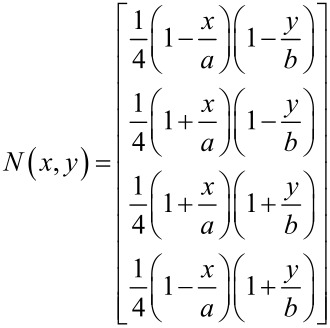


where the dimensions of the rectangular element are 2*a*× 2*b* and the origin is placed at the center of the rectangular element.

By substituting the harmonic solution into Equation 5 the charge response of the piezoelectric transducer over the element is

[12]
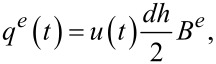


where

[13]
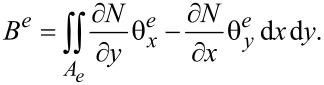


*B^e^* represents the response due to the spatial nature of the piezoelectric layer in the *xy*-plane for a given mode. This expression is evaluated using Gaussian quadrature. Since the shape functions are quadratic, the derivatives are linear. This allows the exact integral to be evaluated with Gaussian quadrature at the midpoint of the rectangular element. Evaluating *B^e^* for each element over the entire cantilever provides the charge response for a given mode.

### Proposed piezoelectric cantilever designs

**Figure 2:**
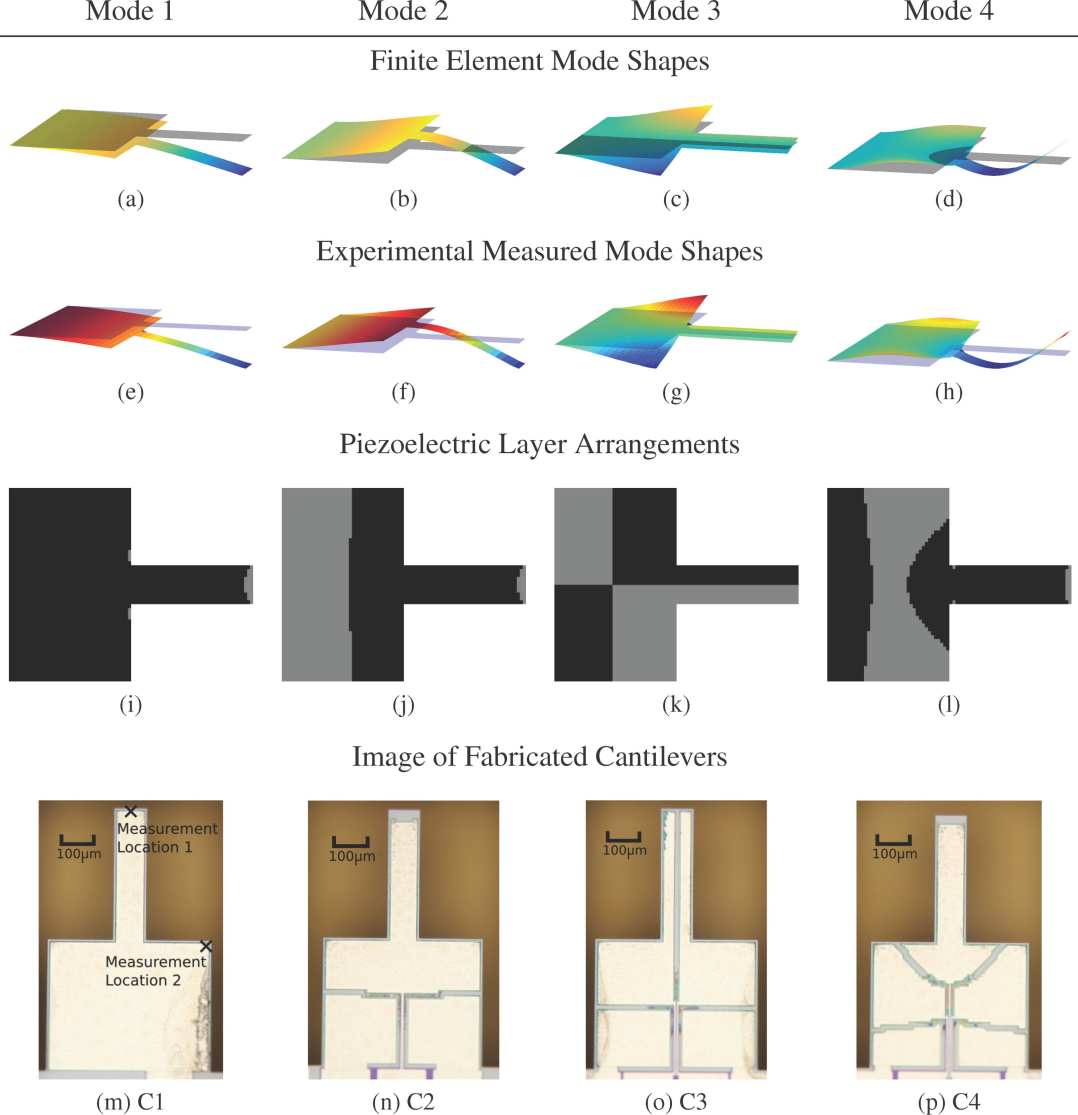
(a)–(d) The first four mode shapes of the cantilever from the FE model. (e)–(f) The modes measured using a laser vibrometer (Polytec MSA-400). (i)–(l) The piezoelectric arrangement to maximize the response of the transducer to each mode. The gray electrodes induce a charge in the opposite polarity to the black electrodes. (m)–(p) The fabricated cantilever designs.

The aim of the proposed cantilever designs is to increase the actuator gain and sensor sensitivity of the piezoelectric transducer to flexural and torsional modes. First, using the finite element method, modal analysis is performed on the cantilever topology shown in Figure 1 to calculate the mode shapes. For mode 1 to mode 4 (M1–M4), the simulated mode shapes of the cantilever are shown in Figure 2a–d. The modal frequencies are 38.3 kHz, 119 kHz, 176 kHz, and 342 kHz. M1, M2 and M4 are flexural modes while M3 is a torsional mode. The modal analysis provides the mode shapes for the deflection and rotations at the nodes of the FE mesh. Using these values, the quantity *B^e^* is calculated for each element in the mesh.

If the sign of *B^e^* for two elements are the same, the response over the two elements adds constructively. If of opposite sign, the response over the two elements adds destructively. Based on the sign of *B^e^*, the piezoelectric layer is split into two, denoted the positive and negative transducer. By actuating and sensing each separately and combining the responses with opposite polarities, the combination of responses is purely constructive. This improves the actuation and sensing by the transducer to its targeted mode.

This design procedure can be posed as an optimization problem. For the *i*-th finite element in the cantilever mesh, the associated *B^e^* is denoted 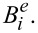 The piezoelectric material on each finite element is observed in either a positive polarity or negative polarity. A design parameter 
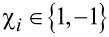
 is introduced indicating this polarity. If χ*_i_* = 1 the finite element is part of the positive transducer otherwise if χ*_i_* = −1 it is connected to the negative transducer. Therefore, maximizing the response of the piezoelectric layer is equivalent to the optimization problem

[14]
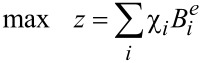


[15]
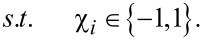


The solution to this optimization problem is χ*_i_* = sign(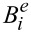). Figure 2i–l shows the split piezoelectric arrangement for the first four modes of the cantilever. In flexural modes, a significant θ*_y_* is observed while θ*_x_* is comparatively small. From Equation 1 and Equation 2 this results in the strain ε*_xx_* dominating the charge response while the effect of ε*_yy_* is insignificant over most of the cantilever area. Though in a few small areas on the cantilever the opposite occurs. In these locations, particularly at the tip of the cantilever, ε*_yy_* dominates the charge response while ε*_xx_* becomes insignificant. This causes the presence of the small electrodes seen in Figure 2i–l.

The fabricated cantilever designs are shown in Figure 2m–p. The cantilevers are fabricated using the PiezoMUMPs microfabrication process available from the company MEMSCAP Inc [54]. The device layer is a 10 μm thick layer of single-crystal-silicon deposited on a (100) oriented wafer. A 0.5 μm layer of AlN and a 1 μm layer of aluminium is deposited on the device layer. A particular limitation of this process in the context of AFM is that it does not allow for the fabrication of tips preventing the demonstration of imaging using these cantilevers.

The material properties of the silicon used in the analysis are an elastic modulus of 169 GPa, density of 2500 kg m^−3^ and Poisson’s ratio of 0.29. To account for inaccuracies in these parameters, the design routine was executed with varying parameters to evaluate their affect on the piezoelectric layer topology. Since the piezoelectric material was not included in the mechanical modeling, the thickness of the silicon layer was also varied. It was found that changes in the mode shape, and thus boundary between the two transducers, was insignificant for densities of 1000–4000 kg m^−3^, elasticities of 120–280 GPa, for Poisson’s ratio of 0.2–0.4 and for a silicon thickness of 8–15 μm. This results from the relative invariance of the mode shape with material properties. Rather, the mode shape is more strongly associated with the geometric shape of the cantilever.

The four cantilevers in Figure 2m–p are denoted C1, C2, C3 and C4. The piezoelectric layer on each cantilever is designed to optimally actuate and sense M1–M4 respectively. Metal traces forming electrical connections run down the center of the cantilever. This splits some piezoelectric layers in two and these split layers are wire-bonded back together.

### Instrumentation of the cantilever

#### Instrumentation design

**Figure 3:**
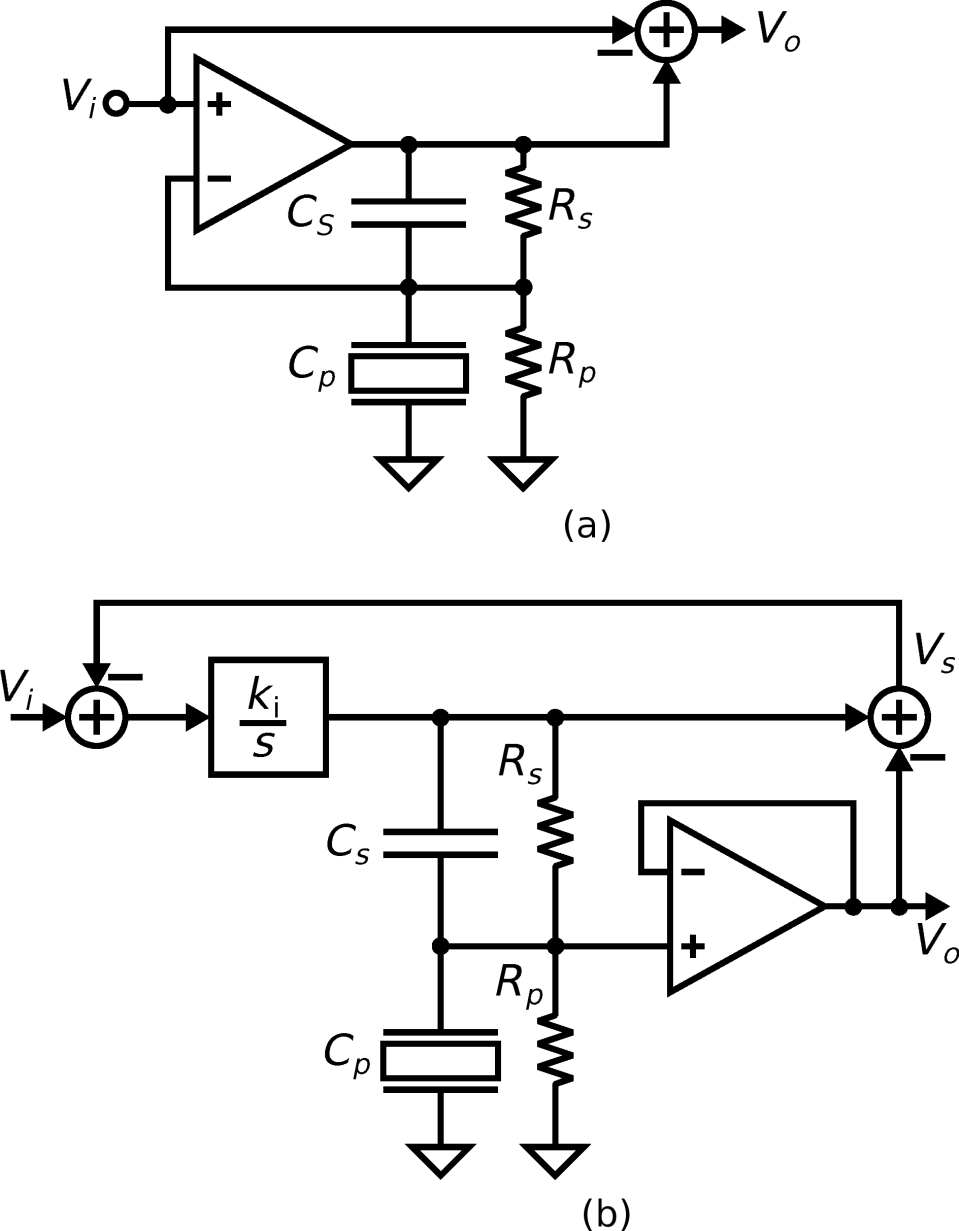
The instrumentation circuits. (a) The voltage driven arrangement. (b) The charge driven arrangement.

The microfabrication process used to fabricate the cantilevers requires the two piezoelectric transducers share a common terminal [54]. The common terminal has to be grounded to electrically isolate them from each other. Therefore, the actuation and sensing circuits are applied to a grounded load. Two instrumentation arrangements are examined, both shown in Figure 3. The first denoted the voltage driven arrangement, is a grounded load charge sensor with the voltage across the device controlled for actuation. The second denoted the charge driven arrangement, is a grounded load charge amplifier and the voltage across the transducer provides the sensor output.

In the voltage driven arrangement [55], an op-amp controls the voltage across the piezoelectric actuator. The charge which flows from the piezoelectric sensor flows into the capacitor *C_s_*. A differential amplifier at the output measures the voltage across *C_s_* to provide a measurement of the charge. The resistors *R_s_* and *R_p_* set the DC biases in the circuit. The FET input op-amp (OPA656 from Texas Instruments) is used to prevent loading of the piezoelectric transducer. The component values used are *C_s_* = 10 pF, *R_s_* = 1 MΩ and *R_p_* = 10 MΩ.

In the charge driven arrangement [56], the circuit controls the charge across the fixed capacitor *C_s_*. An equal charge flows into the piezoelectric transducer as it is in series with *C_s_*. Similar to the voltage driven arrangement, the resistors bias the circuit and the FET op-amp prevents loading of the piezoelectric device. The component values which determine cut-off frequency and gain are chosen as *C_s_* = 100 pF, *R_s_* = 10 MΩ and *R_p_* = 1 MΩ. To prevent oscillations in the instrumentation circuit, an integrator is used to control the charge on *C_s_*. The integrator’s high gain at low frequencies allows the charge to be accurately controlled and their low gain at high frequencies prevents oscillations. The gain of the integral controller *k_i_* is set to maintain *V_i_* = *V_s_* over a bandwidth that contains the modes of interest.

#### Instrumentation modeling

An applied voltage to the piezoelectric transducer excites motion in the cantilever. The mapping from voltage to displacement is modeled as a set of second order modes. The transfer function from voltage *V* to displacement *d* is [52]

[16]
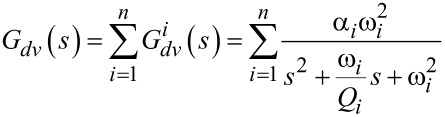


where for the *i*-th mode, ω*_i_* is the natural frequency, *Q_i_* is the quality factor and α*_i_* is the gain.

**Figure 4:**
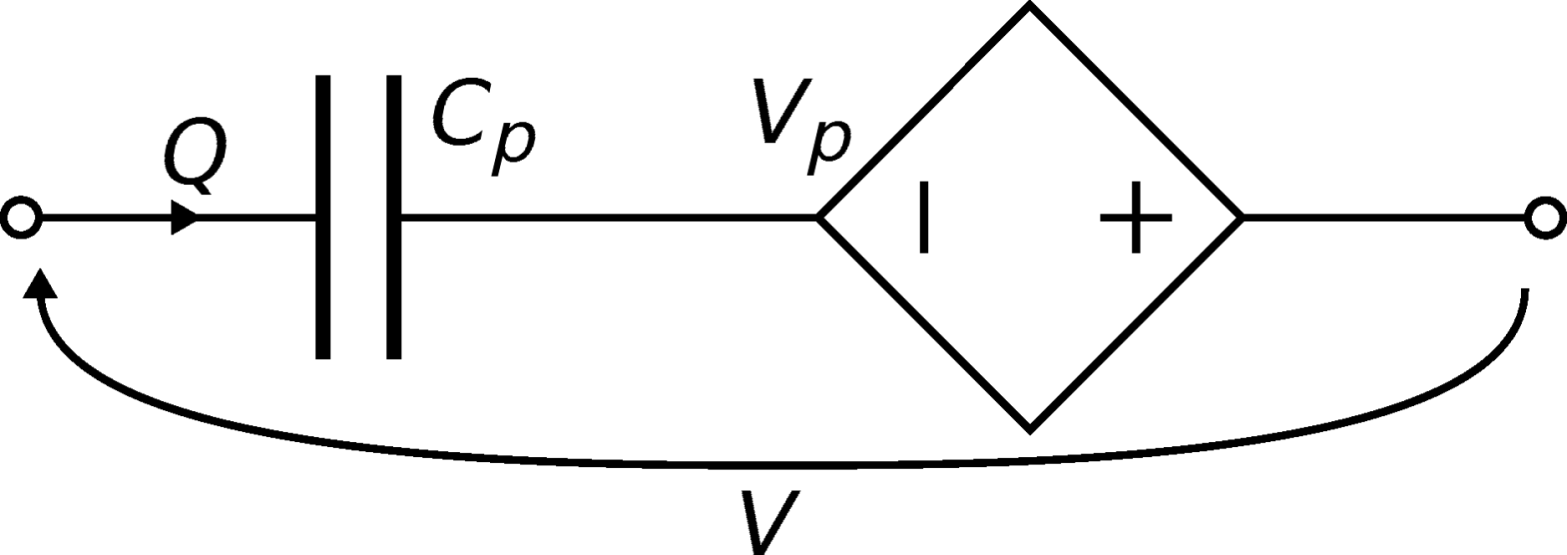
The electrical model of a piezoelectric device.

While under motion, the strain on the piezoelectric transducer induces charge on its electrodes. This effect is modeled as an internal voltage source *V_p_* in series with a capacitor *C_p_* as shown in Figure 4. The transfer function from the applied voltage *V* to the piezoelectric voltage *V_p_* is

[17]
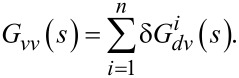


The piezoelectric voltage allows for the electric sensing of the motion of the cantilever. Considering the model in Figure 4, the mapping from the voltage applied to the charge *Q* generated is [22]

[18]
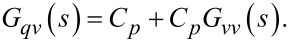


There are two terms in this transfer function. The charge associated with the first term is called the feedthrough charge and the charge associated with the second term is called the motional charge. The feedthrough charge flows due to the capacitive structure of the piezoelectric transducer and the motional charge flows due to the strain and is used to observe the motion of the cantilever.

With the piezoelectric transducer incorporated into the voltage driven circuit, the transfer function of the instrumentation is

[19]
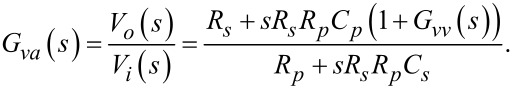


The model can be simplified by considering the dynamics in the neighborhood of the *i*-th cantilever mode. First, the transfer function of the feedthrough component is identified by letting *G_vv_* = 0. The resistance *R_p_* and *R_s_* are chosen such that the pole and zero in the feedthrough transfer function are much lower than the modal frequencies of the cantilever. Then by considering only the frequencies in the passband (i.e., for ω > 1/*R_s_C_s_* and ω > 1/*R_p_C_p_*, *s* = *j*ω), the system *G_va_* becomes

[20]
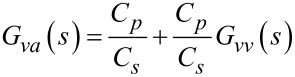


[21]
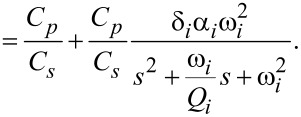


When using the piezoelectric transducer for real-time sensing such as during AFM imaging, the feedthrough component has to be estimated and removed from the sensor response to maximize the dynamic range of the sensor. This can be done by using model-based feedforward compensators, implemented in either analog or using switched capacitor prototyping systems such as a Field Programmable Analog Arrays (FPAAs) [39–40]. Since the cantilevers proposed in this work do not feature tips for AFM imaging, the feedthrough is identified and removed off-line to highlight the increase in sensor sensitivity.

The charge driven arrangement is the inverse of the voltage driven arrangement. The inversion maintains the same structure as in Equation 21, however the resulting transfer function shows flipped poles and zeros (compare Figure 7) as well as slightly differing gains, quality factors and resonance frequencies due to the internal feedback nature in Equation 20 [40]. The transfer function in the neighborhood of the cantilever’s *i*-th mode is

[22]
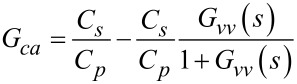


[23]
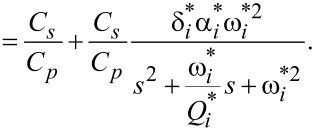


### Experimental results

#### Experimental setup

**Figure 5:**
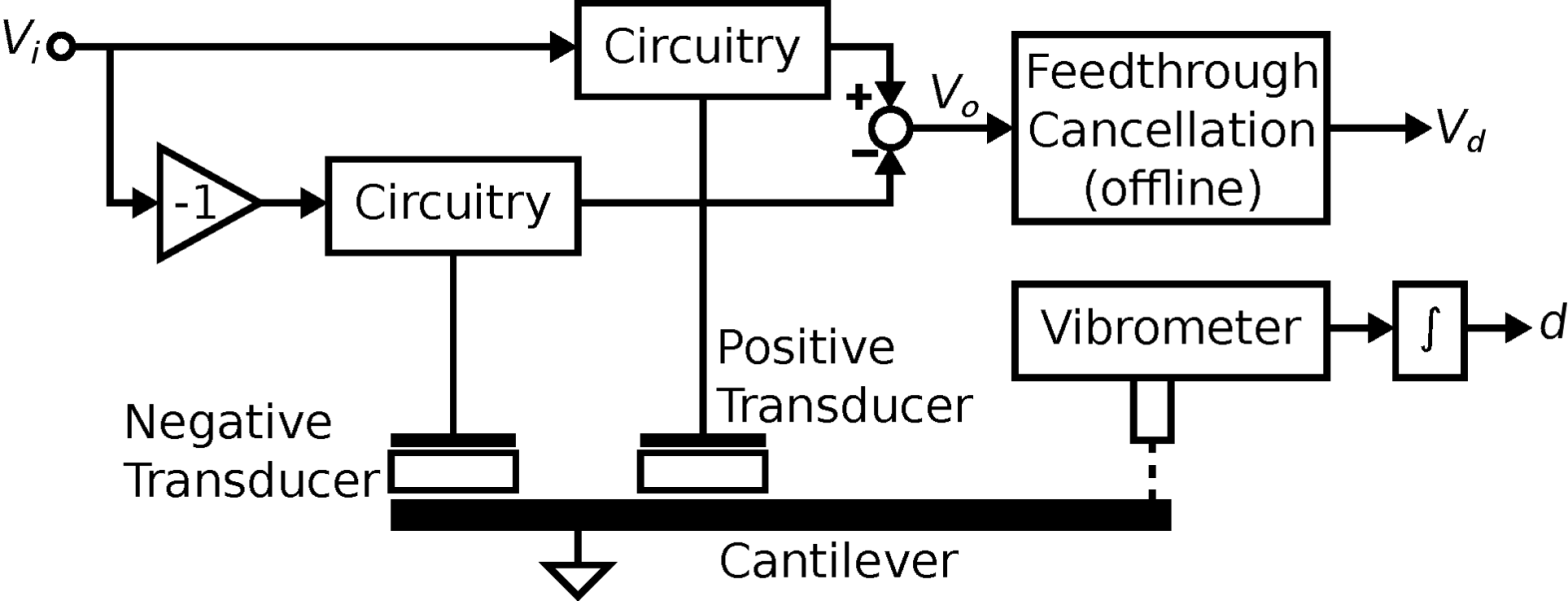
The experimental setup to characterize the cantilever designs.

Figure 5 shows the experimental setup to characterize the performances of cantilever designs (C1 to C4). The positive and negative transducers are connected to separate instrumentation circuits which are actuated and sensed in the opposite polarity to constructively combine the two responses. For displacement measurement, a vibrometer (Polytec MSA-400) is used to detect the motion of the cantilever. The actuator gains (*V_i_*→*d*) are measured in two locations which are shown in Figure 2m. Measurements are made at location 1 for flexural modes M1, M2 and M4 and location 2 for the torsional mode M3.

The system response (*V_i_*→*V_o_*) is the combination of a motional and feedthrough component. To observe the motional component feedthrough cancellation is performed offline. A third order transfer function is fitted to the measured frequency response in a small band around the mode of interest. The third-order model accounts for a second order mechanical system with a first order feedthrough system in parallel. Identification is performed using the subspace method [57]. The identified feedthrough is subtracted from the measurements to produce the system response with feedthrough cancellation (*V_i_*→*V_d_*). Due to small phase shifts from the op-amp dynamics and unmodeled electrical parasitics, feedthrough cancellation can only be accurately performed in a narrow-band in the vicinity of the mode of interest.

To evaluate the effect of the proposed piezoelectric topologies, the magnitude responses from *V_i_* to both *V_d_* and *d* are measured for each cantilever and compared to the response of C1. C1 is used as the reference cantilever because it is considered a standard topology with the piezoelectric layer covering the entire cantilever.

#### Discussion of results

**Figure 6:**
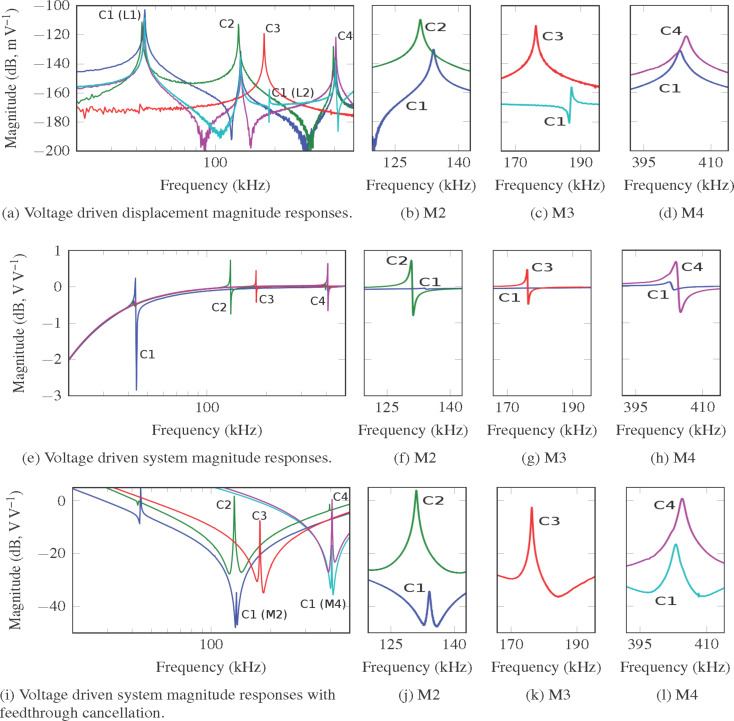
Voltage drive magnitude responses of the cantilevers C1–C4. (a)–(d) The responses from input voltage *V_i_* to displacement *d*. The flexural modes of C1, C2, and C4 are measured at location 1 (L1). The torsional modes of C1 and C3 are measured at location 2 (L2) (see Figure 2m). (e)–(h) The responses from *V_i_* to output voltage *V_o_*. To show the resonance more clearly, the plots for C1–C4 are moved to 0 dB with a shift of −15.21 dB, −18.85 dB, −16.6 dB and −16.57 dB respectively to account for the affect of different values of *C_p_* in each cantilever. (i)–(l) The responses from *V_i_* to sensor output *V_d_*. (b)–(d), (f)–(h) and (j)–(l) show higher resolution plots of the modes.

From the magnitude frequency response of C1 (Figure 6a), the frequency of the cantilever’s first four modes are at 44.02 kHz, 133.7 kHz, 186.8 kHz and 402.9 kHz respectively. Fabrication tolerances and the mechanical action of the piezoelectric layer account for the frequency differences compared to the FE model in section ’Proposed piezoelectric cantilever designs’.

**Figure 7:**
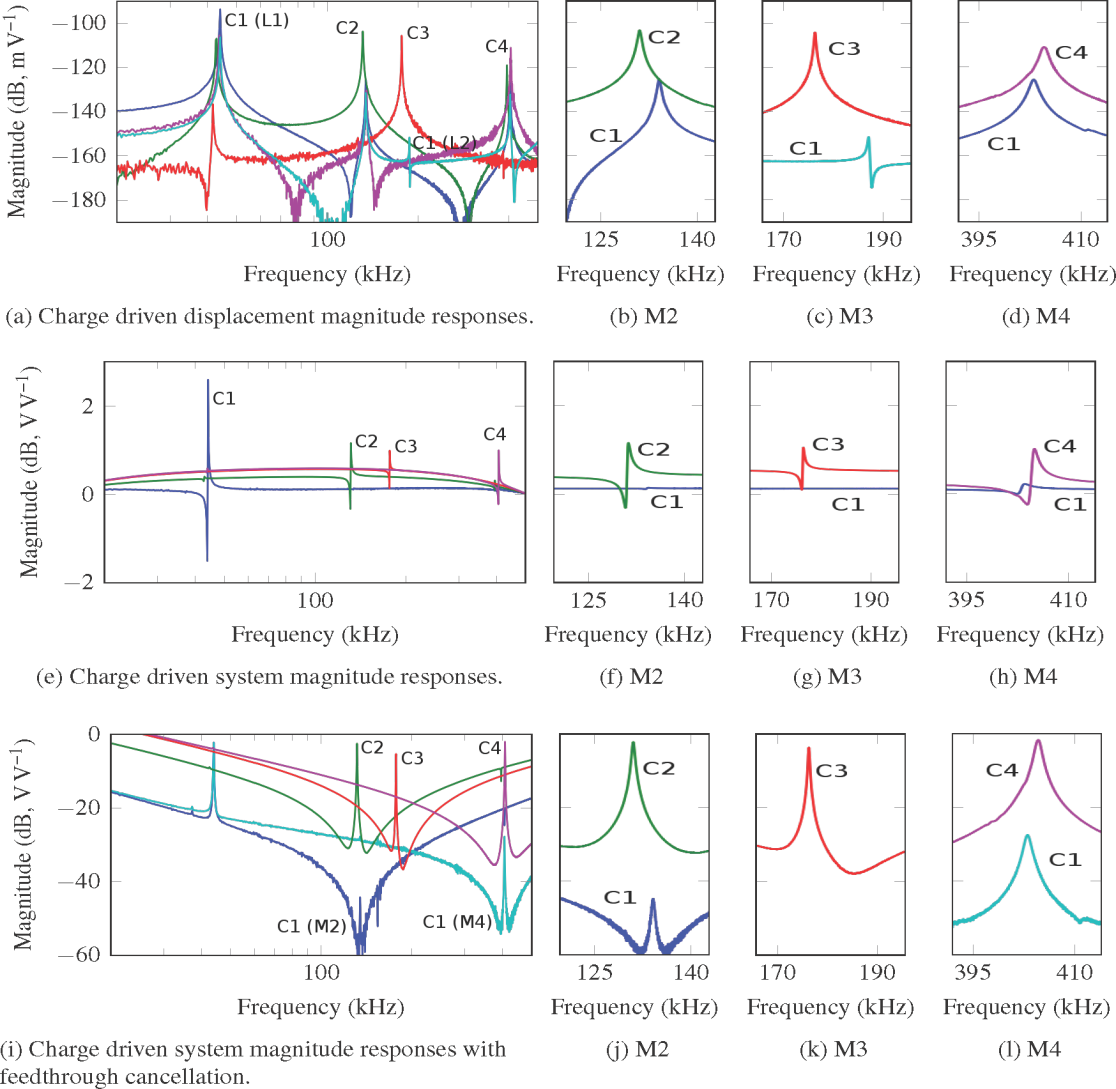
Charge driven magnitude responses of the cantilevers C1–C4. (a)–(d) The responses from input voltage *V_i_* to displacement *d*. The flexural modes of C1, C2, and C4 are measured at location 1 (L1). The torsional modes of C1 and C3 are measured at location 2 (L2) (see Figure 2m). (e)–(h) The responses from *V_i_* to output voltage *V_o_*. To show the resonance more clearly, the plots for C1–C4 are moved to 0 dB with a shift of −3.984 dB, −12.42 dB, −14.46 dB and −14.50 dB respectively to account for the affect of different values of *C_p_* in each cantilever. (i)–(l) The responses from *V_i_* to sensor output *V_d_*. (b)–(d), (f)–(h) and (j)–(l) show higher resolution plots of the modes.

In Figure 6a–d (voltage driven) and Figure 7a–d (charge driven), the frequency responses from the input voltage *V_i_* to the displacement *d* are shown. The magnitude of the response at each mode is tabulated in Table 2a and c. Compared to C1 in the voltage driven arrangement, C2 provides a 20.45 dB increase in actuation gain for mode 2. C3 provides a 42.05 dB increase in actuation gain for mode 3. C4 provides an 10.04 dB increase in gain for mode 4. Compared to C1 for the charge driven arrangement C2 provides a 22.04 dB increase in actuation gain for mode 2. C3 provides a 47.01 dB increase in actuation gain for mode 3. C4 provides an 14.56 dB increase in gain for mode 4.

**Table 2:** Sensitivities of the reference cantilever C1 compared to the cantilevers with optimal transducer topologies.

(a) Voltage driven actuator gain (*V_i_*→*d*).			
mode	Reference gain [μm V^−1^]	Optimized gain [μm V^−1^]	Improvement [dB]

M1	(C1) 16.97	–	–
M2	(C1) 2.968 × 10^−1^	(C2) 3.125	20.45
M3	(C1) 1.522 × 10^−2^	(C3) 1.927	42.05
M4	(C1) 2.667 × 10^−1^	(C4) 8.475 × 10^−1^	10.04

(b) Voltage driven magnitude of system responses with feedthrough cancellation (*V_i_*→*V_d_*).			
mode	Reference gain [V V^−1^]	Optimized gain [V V^−1^]	Improvement [dB]

M1	(C1) 3.265	–	–
M2	(C1) 1.910 × 10^−2^	(C2) 1.514	37.98
M3	–	(C3) 7.247 × 10^−1^	–
M4	(C1) 1.471 × 10^−1^	(C4) 1.061	17.16

(c) Charge driven actuator gain (*V_i_*→*d*).			
mode	Reference gain [μm V^−1^]	Optimized gain [μm V^−1^]	Improvement [dB]

M1	(C1) 25.08	–	–
M2	(C1) 5.034 × 10^−1^	(C2) 6.373	22.04
M3	(C1) 2.626 × 10^−2^	(C3) 5.890	47.01
M4	(C1) 4.999 × 10^−1^	(C4) 2.673	14.56

(d) Charge driven magnitude of system responses with feedthrough cancellation (*V_i_*→*V_d_*).			
mode	Reference gain [V V^−1^]	Optimized gain [V V^−1^]	Improvement [dB]

M1	(C1) 9.385 × 10^−1^	–	–
M2	(C1) 5.687 × 10^−3^	(C2) 7.423 × 10^−1^	42.31
M3	–	(C3) 6.251 × 10^−1^	–
M4	(C1) 4.128 × 10^−2^	(C4) 7.910 × 10^−1^	25.65

In Figure 6e–h and Figure 7e–h, the frequency responses from input voltage *V_i_* to output voltage *V_o_* are shown. Here substantial feedthrough is observed which dominates in comparison to the motional response of the system. In a neighborhood around the modes of interest, a third order system is identified. Transfer functions were identified around C1 mode 2, C1 mode 4, C2 mode 2, C3 mode 3 and C4 mode 4. The motional response for C1 mode 3 was unobservable due to the small magnitude of resonance response. The parameters of Equation 21 and Equation 23 for each of these transfer functions are tabulated in Table 3.

**Table 3:** Parameters of the identified transfer functions around the modes.

(a) Voltage driven system parameters.				
	*C_p_*/*C_s_*	*Q_i_*	*f_i_* [kHz]	δ*_i_*α*_i_*

C1 (M1)	5.433	408.6	43.92	1.395 × 10^−3^
C1 (M2)	5.716	316.6	133.5	9.630 × 10^−6^
C1 (M4)	5.756	358.9	402.9	7.074 × 10^−5^
C2 (M2)	8.698	309.0	130.7	5.545 × 10^−4^
C3 (M3)	6.718	608.9	175.9	1.761 × 10^−4^
C4 (M4)	6.728	381.0	404.3	4.144 × 10^−4^

(b) Charge driven system parameters.				
	*C_s_*/*C_p_*	Q_i_^*^	f_i_^*^ [kHz]	δ_i_^*^α_i_^*^

C1 (M1)	1.599	390.3	43.94	1.506 × 10^−3^
C1 (M2)	1.602	277.4	133.8	1.260 × 10^−5^
C1 (M4)	1.598	371.1	402.9	6.562 × 10^−5^
C2 (M2)	4.370	290.3	130.7	5.811 × 10^−4^
C3 (M3)	5.613	578.7	176.0	1.869 × 10^−4^
C4 (M4)	5.434	386.1	404.4	3.077 × 10^−4^

The resulting magnitude responses with the feedthrough cancellation from input voltage *V_i_* to sensor output *V_d_*, are shown in Figure 6i–l (voltage driven) and Figure 7i–l (charge driven). Around each mode the feedthrough is removed and the motional component in the neighborhood of the modal frequency is revealed. The magnitudes of the motional components at each modal frequency are tabulated in Table 2b,d. For the voltage driven arrangement C2 increases the system response of mode 2 by 37.98 dB. C4 increases the system response to mode 4 by 17.16 dB. For the charge driven arrangement C2 increases the system response of mode 2 by 42.31 dB. C4 increases the system response to mode 4 by 25.65 dB.

#### Noise discussion

**Figure 8:**
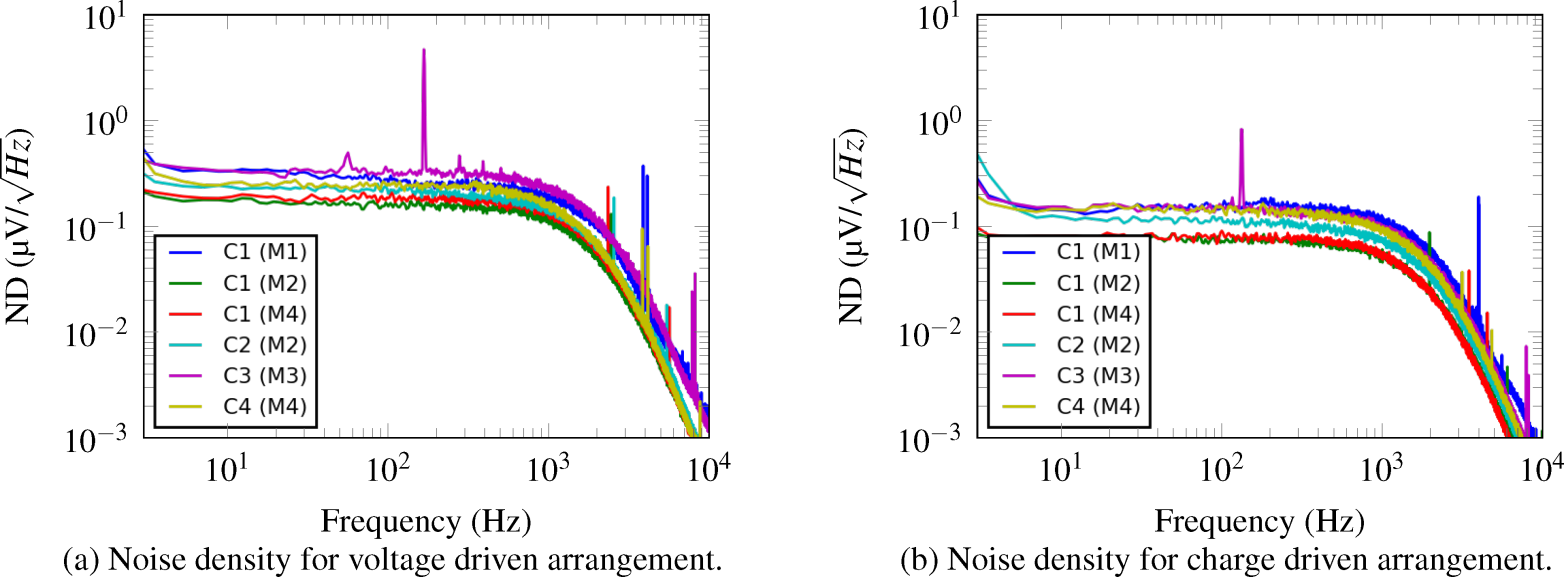
Amplitude noise density spectra of the output voltage *V_o_* for the reference cantilever C1 and optimized cantilevers C2–C4. (a) Voltage driven instrumentation, (b) charge driven instrumentation.

Amplitude modulation AFM always requires an actively driven cantilever and subsequent demodulation using a lock-in amplifier. Therefore, the subsequent noise characterization is for the demodulated amplitude signal. A 4th-order low-pass filter with cut-off frequency *f_c_* = 1 kHz is employed in the lock-in amplifier (Zurich Instruments, HF2LI). The voltage noise density plot of the sensor output is obtained by sampling the demodulated amplitude at *f_s_* = 57.6 kHz and calculating a power spectral density estimate using Welch’s segment averaging estimator with 64 segments. The results are presented in Figure 8.

The RMS voltage noise present in the sensor output voltage is obtained by integrating the noise density (Figure 8) from 0 to *f_s_*/2. The deflection noise is obtained by dividing the voltage noise by the identified sensor sensitivity. The sensor sensitivities are obtained by dividing the system response (Table 2b,d) by the actuator gain (Table 2a,c) for each mode. The voltage noise measurements are made before the feedthrough cancellation, therefore the deflection noise results are for the case where the feedthrough cancellation is noiseless. The sensor sensitivities, sensor output voltage noise and sensor output deflection noise are tabulated in Table 4.

**Table 4:** Sensor sensitivities and noise performance of non-optimized and optimized higher order modes.

(a) Voltage driven arrangement.						
	C1 M1	C1 M2	C1 M4	C2 M2	C3 M3	C4 M4

Sensor sensitivity [V/μm]	0.192	0.0644	0.551	0.484	0.376	1.25
RMS voltage noise [μV]	9.6959	5.4284	6.2378	7.0169	13.9297	8.2834
RMS deflection noise [pm]	50.4	84.3	11.3	14.5	37.0	6.62

(b) Charge driven arrangement.						
	C1 M1	C1 M2	C1 M4	C2 M2	C3 M3	C4 M4

Sensor sensitivity [V/μm]	0.0374	0.0113	12.11	8.586	9.422	3.379
RMS voltage noise [μV]	5.7846	2.5007	2.6202	5.4924	5.3145	4.5816
RMS deflection noise [pm]	154.6	221.4	31.73	47.16	50.07	15.48

Due to the higher sensor sensitivities, the deflection noise has decreased demonstrating an improved sensor performance resulting from shaping the piezoelectric layer. This result occurs when noise sources from the sensor and instrumentation dominate the noise output rather than thermomechanical noise. Thermomechanical noise is amplified by the sensor sensitivity and since an associated increase in noise was not observed, this noise source must be insignificant compared to the noise from the instrumentation.

#### Instrumentation for multifrequency AFM

**Figure 9:**
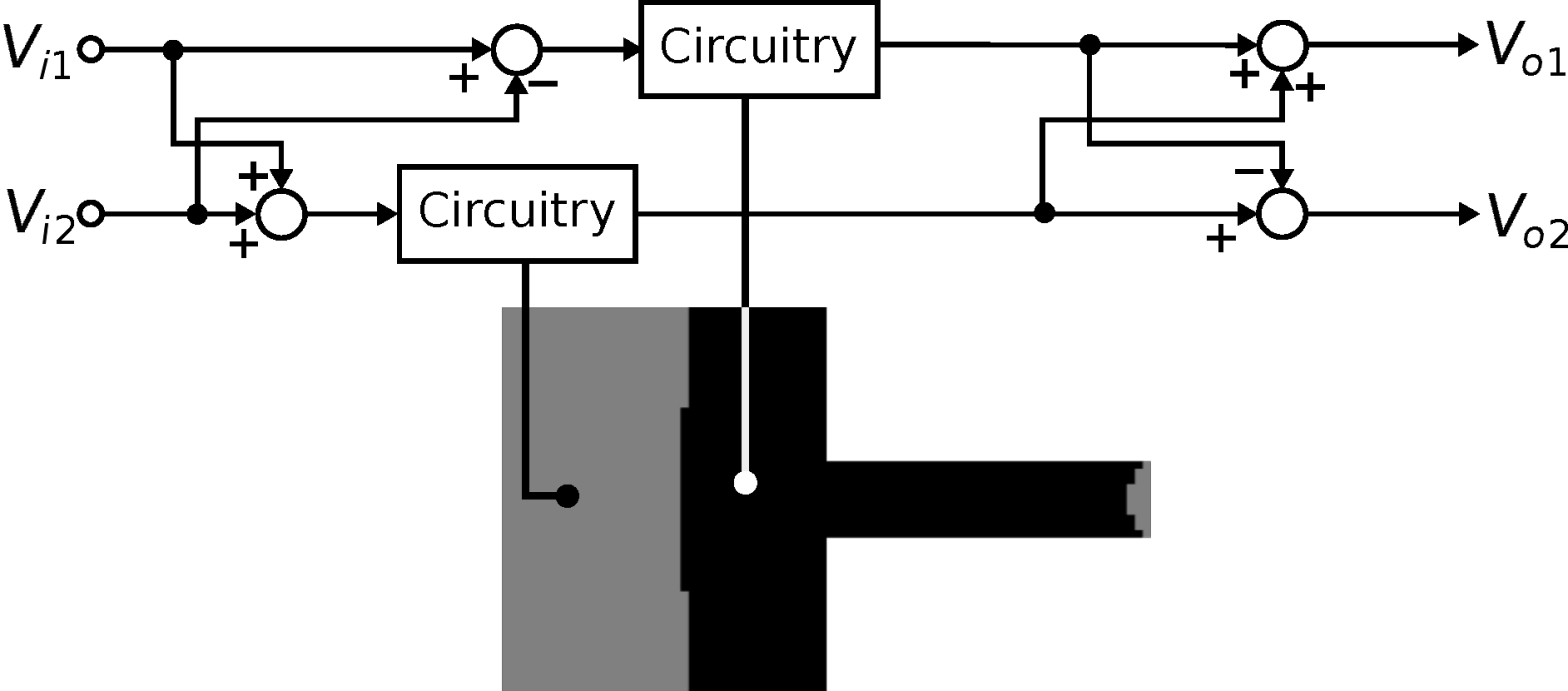
To perform multifrequency AFM using cantilever C2 with modes M1 and M2 simultaneously, the presented extension to the instrumentation is employed.

The cantilevers fabricated and characterized in this work each enhance the sensitivity of a single mode. To facilitate multifrequency AFM, it is preferable to have a cantilever optimized to actuate and sense multiple modes and in addition allow them to be transduced simultaneously with maximized sensitivities. Given the linear nature of the cantilever and instrumentation, superposition can be used to achieve this. For example, the cantilever C2 has the potential to optimally actuate and sense modes M1 and M2 simultaneously. In the setup shown in Figure 9, the M1 input voltage *V_i1_* is driven at the M1 resonance frequency and is applied to both piezoelectric transducers with the same polarity to optimally actuate the cantilever for M1. The outputs of the two instrumentation circuits are combined with the same polarity to produce the optimized M1 output voltage *V_o1_* which maximizes the sensed response for M1. The M2 input voltage *V_i2_* is combined with *V_i1_*, however, for the second transducer *V_i2_* is combined with a negative polarity to optimally actuate M2. The optimal output voltage for M2, *V_o2_*, results from taking the difference between the outputs of the two instrumentation circuits.

**Figure 10:**
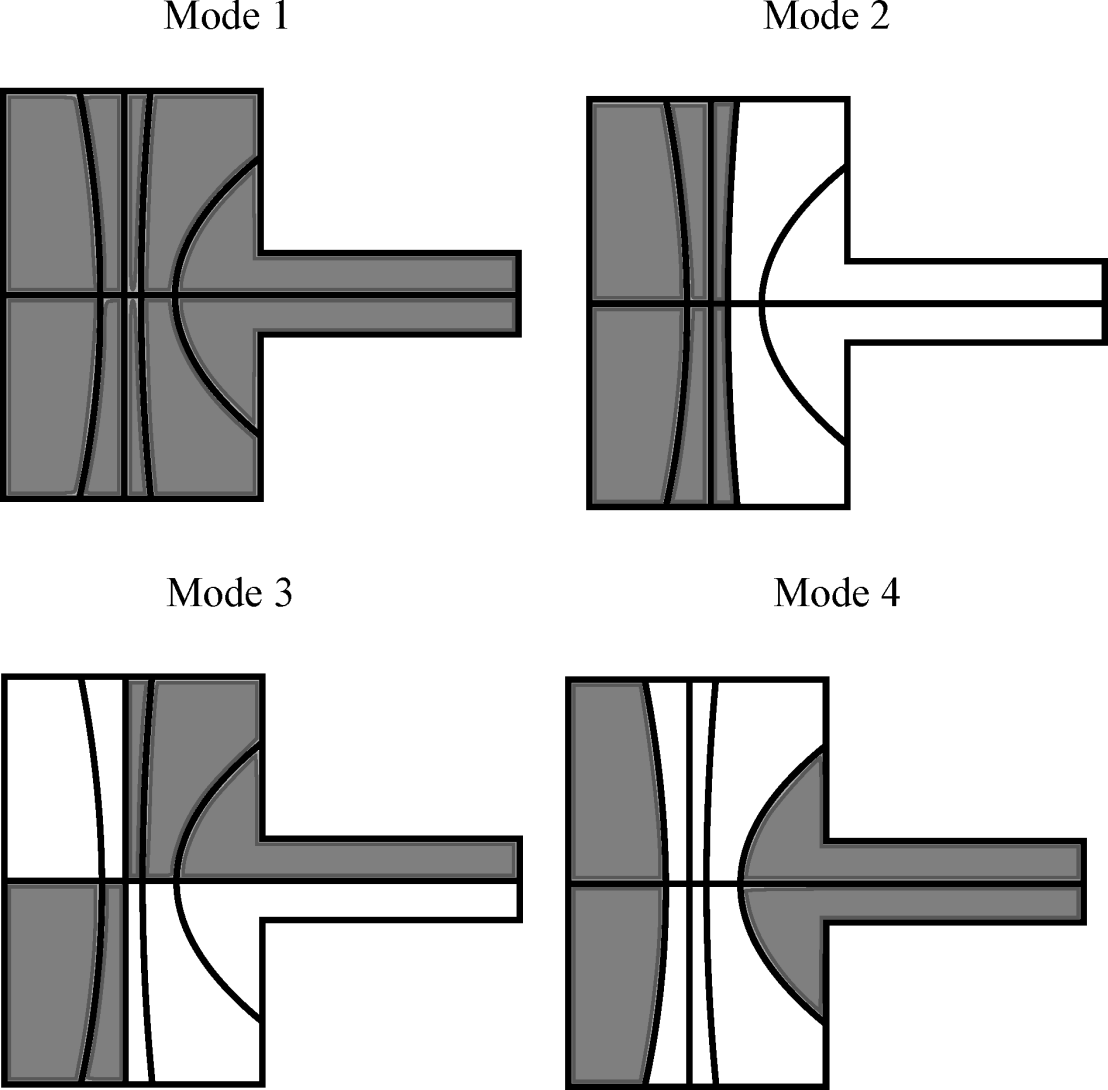
The layout of the piezoelectric layer on the cantilever to optimally actuate and sense M1–M4. The cantilever has 10 separate transducers. The voltage on each transducer is a combination of four voltages to actuate each mode. These four voltages are combined in the positive polarity for the black electrodes and in the negative polarity for white electrodes. The sensor outputs for each transducer are combined in the same fashion for each mode.

This principle can be extended for the design of a piezoelectric cantilever used to actuate and sense multiple modes. The design example for the first four modes is shown in Figure 10. By considering the union of cantilevers C1–C4, the resulting cantilever has 10 separate piezoelectric transducers each with its own instrumentation circuit. The M1–M4 input voltages *V_i1_*–*V_i4_*, each driven at their respective modal resonance frequencies, are applied to the cantilever on each transducer with a positive polarity for black electrodes (refer to Figure 10) and a negative polarity for white electrodes. The outputs of the instrumentation circuits would be combined in the same fashion for the optimized output voltages *V_o1_*–*V_o4_*. In comparison to using a single piezoelectric transducer with a sum of sinusoids excitation, the multi-electrode design provides increased amplitudes at the expense of more complex instrumentation.

## Conclusion

An AFM cantilever with a piezoelectric layer is a versatile transducer for both actuation and displacement sensing. As the response of the active layer is a function of the strain over the surface of the cantilever, careful electrode layout has to be employed. Specifically in multifrequency AFM, the cantilever is excited at higher order modes for which the spatial distribution of the strain cause portions of the piezoelectric layer to contribute destructively to the transducer’s overall response. In this work, we have outlined a design method which provides a systematic way of increasing the actuator gain and sensor sensitivity of the self-sensing piezoelectric cantilever by considering the spatial distribution of the strain for a given mode. The design consists of splitting up the piezoelectric layer into several transducers, whose individual responses are constructively combined. The resulting three terminal piezoelectric device requires additional circuitry for instrumentation compared to a typical two terminal piezoelectric cantilever. For this reason, we have proposed a grounded load charge sensor and a grounded load charge amplifier to realize the self-sensing implementation. The experimental results show that by shaping the electrodes on the piezoelectric layer, significant increases in actuator gain and sensor sensitivity are attained. Furthermore, torsional modes are strongly observed in contrast to a cantilever with an evenly distributed piezoelectric layer. Despite the additional circuitry, no significant increase in noise was observed. Future work will focus on the fabrication of cantilevers with tips with the aim of exploiting the proposed technique to potentially perform higher precision imaging in multifrequency AFM.
